# Role of Sugar and Sugar Substitutes in Dental Caries: A Review

**DOI:** 10.1155/2013/519421

**Published:** 2013-12-29

**Authors:** Prahlad Gupta, Nidhi Gupta, Atish Prakash Pawar, Smita Shrishail Birajdar, Amanpreet Singh Natt, Harkanwal Preet Singh

**Affiliations:** ^1^Department of Public Health Dentistry, Dasmesh Institute of Research and Dental Sciences, Faridkot, Punjab, India; ^2^Department of Prosthodontics, Dasmesh Institute of Research and Dental Sciences, Faridkot, Punjab 151203, India; ^3^Department of Oral Pathology, Aditya Dental College, Beed, Maharashtra 431122, India; ^4^Department of Oral Pathology, Vishnu Dental College and Hospital, Bhimavaram 534 202, India; ^5^Department of Orthodontics, Dasmesh Institute of Research and Dental Sciences, Faridkot, Punjab, India; ^6^Department of Oral Pathology and Microbiology, Dasmesh Institute of Research and Dental Sciences, Faridkot, Punjab, India

## Abstract

Dental caries is a chronic disease which can affect us at any age. The term “caries” denotes both the disease process and its consequences, that is, the damage caused by the disease process. Dental caries has a multifactorial aetiology in which there is interplay of three principal factors: the host (saliva and teeth), the microflora (plaque), and the substrate (diet), and a fourth factor: time. The role of sugar (and other fermentable carbohydrates such as highly refined flour) as a risk factor in the initiation and progression of dental caries is overwhelming. Whether this initial demineralization proceeds to clinically detectable caries or whether the lesion is remineralized by plaque minerals depends on a number of factors, of which the amount and frequency of further sugars consumption are of utmost importance. This paper reviews the role of sugar and sugar substitutes in dental caries.

## 1. Introduction

To most people the term “sugar” refers to the common household foodstuff table sugar (sucrose). Yet sucrose is only one of many naturally occurring sugars used in the human diet. Technically the term “sugars” applies to two classifications of carbohydrates: free-form monosaccharides (simple sugars) which include the more common glucose, fructose, and galactose and disaccharides (two simple sugar molecules linked together) which include the most common sucrose, lactose, and maltose. Naturally occurring sugars are available in fruits, vegetables, grains, and dairy foods. Sweeteners are added sugars that are used as ingredients to both satisfy our taste and in some cases provide added energy. Grouping sweeteners as “nutritive” or “nonnutritive” acknowledges a difference in the amount of energy provided by the sweetener. Nutritive sweeteners may be referred to as caloric and include sugars and sugar alcohols. Nonnutritive sweeteners offer no energy and can sweeten with little volume. Both sugar alcohols and nonnutritive sweeteners can replace the sugars and are sometimes referred to as sugar substitutes, sugar replacers, or alternative sweeteners [1]. We searched the pertinent literature in PubMed and MEDLINE databases by using key words such as sugar in dental caries, sugar substitute in dental caries, and various types of sugar in dental caries.

## 2. Sugar and Dental Caries

Of the many factors that contribute to the development of dental caries, diet plays an important role. Fifty years ago dietary issues relevant to dental caries were largely concerned with dietary sugars. Although sugars are undoubtedly the most important dietary factors in the etiology of dental caries, today's diet contains an increasing range of fermentable carbohydrates, including highly processed starch-containing foods and foods that contain novel synthetic carbohydrates such as oligofructose, sucralose, and glucose polymers. Coupled with this, there now exists a wide range of noncariogenic sweeteners that have an important role to play in caries control. Research continues to identify foods and factors that protect against dental caries and those that have a practical dietary application and can help to make dietary advice more positive, which aids compliance [2].

## 3. Cariogenicity of Different Sugars

Sucrose for years was billed as the “archcriminal” of dental caries because it was considered to be so much more cariogenic than other sugars [3]. However, later research has suggested that the differences between sucrose and the various monosaccharides in terms of cariogenic potential are less than originally believed [4, 5]. This is a difficult issue to study in humans because of the variability of the human diet, so views are based principally on extrapolations from animal studies and laboratory research. One study in Sweden involving a small number of preschool children found that those consuming invert sugar (a mixture of glucose and fructose) in place of sucrose had a lower caries increment in 2 years, although the differences did not reach statistical significance [6]. However, one could speculate whether reduced consumption of sucrose in the developed countries has been a factor in the sharp reduction in approximal and smooth surface caries relative to the overall caries decline. This speculation is based on the fact that the production of extracellular polysaccharides in plaque depends on sucrose and that smooth surface caries will only develop with plaque that adheres by means of extracellular polysaccharides [7].

Sugars can be readily metabolized by many bacteria involved in dental biofilm formation, generating acid byproducts that can lead to demineralization of the tooth structure. Lactose (milk sugar) has been shown to be less acidogenic than other sugars and less cariogenic, based on animal studies [8]. Sucrose has been given special consideration as a cariogenic substrate owing to its unique ability to support the synthesis of extracellular (water-soluble and water-insoluble) glucans by mutans streptococci, enhancing its accumulation in the plaque. Some animal studies on rats superinfected with *Streptococcus miutans *have reported increased cariogenicity of sucrose compared to other sugars; however, this effect appears to be bacterial strain-specific and not consistent across different animal models. More recent clinical studies have indicated that the caries-associated virulence of glucan may have more to do with an alteration in plaque ecology by increasing the porosity of plaque, permitting deeper penetration of dietary sugars and greater acid production adjacent to the tooth surface [9].

## 4. Influence of Different Sugar Intake Patterns: Amount, Form, and Frequency

It is generally accepted that the prevalence of caries is related to the form in which sugar is ingested and the frequency of its consumption. By “form” we meant the physical consistency of the sugar-containing foods. Distinctions are made between liquid and adhesive (sticky) foods as well as foods which vary in adhesiveness between the extremes. The term “frequency” refers to the number of times per day that sugary foods are eaten. It is clear that both form and frequency affect the length of time that teeth are exposed to sugar [10]. However, the relative importance of frequency versus the total amount of sugar consumption is difficult to evaluate.

The relationship of the physical consistency of food to caries is not entirely clear. Several studies have incriminated the stickiness of foods as prime factor in the initiation of caries [11, 12]. Others have shown that semisolid and even liquid sugar-containing foods can be very cariogenic [13]. Ericsson [14] reported that the frequent intake of lozenges can cause rampant decay, so can liquids as is evident in the case of nursing bottle caries [15, 16] and in experimental human studies investigations by von der Fehr et al. [17] and Geddes et al. [18]. It is likely that the length of time that the teeth are exposed to sugar-containing foods rather than simply the form of the food is a critical factor in the promotion of caries [10]. Many studies point to the frequency of eating sugars to be of greater etiological importance for caries than the total consumption of sugars [19, 20]. The primary evidence comes from the Vipeholm study [11]. A positive correlation between the frequency of consumption of confectionery and sugar-containing gum and the DMF rate was also found in a study conducted on 14-year-old Caucasian, Hawaiian, and Japanese schoolchildren in Hawaii [21]. A range in intake from zero to five or more sweets per day was followed by a corresponding increase in DMF scores. Against the general perception that frequency of intake is more important than the amount of sugars eaten, two longitudinal studies reported the amount of sugars intake to be more important than frequency [8, 22, 23]. However, there is undoubtedly a strong correlation between the two variables [8] with an increase in one factor often leading to an increase in the other.

Although a high intake frequency increases the overall length of time that the teeth are exposed to sugars, it does not give complete information on the total time of exposure. The total cariogenic load is also determined by the form of the food product; that is, the physical consistency of the sugar-containing foods affects their retention time in the mouth. Distinctions can be made between liquids that are cleared rapidly and adhesive (sticky) foods that vary widely in retentiveness. Particularly high retention rates have been found for products such as sweet biscuits, crackers, and potato chips (crisps) [24]. Other aspects of intake pattern are also believed to be of importance. The sequence of eating a cariogenic food product during a meal or snack can alter its cariogenic properties. Both cheese and peanuts can reduce the acid production after a previous intake of sucrose-containing foods. Conversely, starches can increase the cariogenic properties of sugars if they are consumed at the same time. The stickiness of starch enhances the retention time of sugars, resulting in a prolonged pH fall, as occurs in breakfast cereals with added sugars. Another important issue that is difficult to account for in determining the relationship between the dietary intake and caries is that many food products contain hidden sugars. Examples of such sugar-containing products may vary from one country to another. It is not obvious to most people that sugars may be a major constituent in products such as marmalade, breakfast cereals, flavored crisps (chips), caviar, ketchup, and, in many countries, bread. Thus, just focussing on confectioneries may have little impact on reducing caries activity if an individual is exposed to many other sugary products per day [8].

## 5. Oral Clearance of Sugars

Saliva plays a major role in protecting the teeth, owing to its cleaning actions as well as its acid neutralizing, antisolubility, and antimicrobial properties. A high secretion rate, together with mastication, helps to eliminate sugars and food particles from the oral cavity. A short clearance time reduces the length of time that sugar is available for acid production by the bacteria in the dental plaque. During hyposalivation, which can be found in relation to irradiation in the head and neck area, Sjogren's syndrome, surgery or medication, or in older age groups with poor health, an increased caries rate is often found. Lanke [25] performed studies on the clearance of sugar from saliva to relate the intake of sugar to its availability for bacterial degradation. It was demonstrated that food factors such as sugar concentration, rate of solubilization, rate of enzymatic degradation, ability to adhere to the teeth, and ability to stimulate salivary flow all affected the rate at which sugar is cleared from saliva. More recent studies have supported this view, revealing slower clearance rates resulting in increased risk for caries in the elderly [26] during an artificially induced low secretion rate [27] and for individuals with normally low secretion rates [28]. The clearance rate is believed to be of great importance for today's elderly population, where medically induced low secretion rates are often found in combination with a high number of teeth remaining well into old age. This is of particular importance for the relationship of diet to root caries [29]. In all age groups, different medical conditions such as depression, eating disorders, dementia, and malnutrition may directly, or via medication, influence salivary properties, resulting in increased caries rates.

## 6. Sugar Substitutes and Dental Caries

The development of noncaloric sugar substitutes, marketed for weight control, is big business in the United States. Commercial development of these products, from the laboratory to marketing, is time consuming and expensive. Aspartame, a dipeptide composed of two naturally occurring amino acids, became available in the United States in 1982 [30]. One of the main conclusions from the aforementioned Vipeholm study [11] was that sugars in sticky foods consumed between meals was associated with high caries activity. These findings stimulated research on nonacidogenic sugar substitutes (sweeteners) that do not cause pH falls in dental plaque [31]. It was not until 20 years later, however, that systematic studies carried out in Europe on alternate sweeteners for caries control were published [32–34].

It is imperative to remember that the usefulness of a sugar substitute has to be looked upon not only from a cariological but also from a nutritional, toxicological, economic, and technical point of view. When evaluating a nonsugar sweetener in relation to dental caries, it is important to consider the potential for metabolism by oral microorganisms and dental plaque, the influence of consumption on cariogenic microorganisms, and the risk of microbial adaptation to the sweetener. Sugar substitutes can be categorised into two major groups: intense sweeteners (noncaloric) like aspartame, saccharin, sulfame, glycyrrhizin, and so forth and bulk sweeteners (caloric) like sorbitol, xylitol, mannitol, and so forth [30]. Intense sweeteners are not metabolized to acids by oral microorganisms; thus they cannot cause dental caries. However, it is important to remember that other ingredients, such as citric or phosphoric acids in beverages, may cause dental erosion. In some food products, intense sweeteners are added as well as sugars, for example, to fruit-flavored soft drinks, and the naturally occurring sugars in the drink (fructose, glucose, and sucrose) may cause caries [35]. One of the disadvantages of the bulk sweeteners is that they are only partially absorbed in the small intestine and thus may induce osmotic diarrhea [36]. For this reason food and drinks containing bulk sweeteners are not recommended for children under 3 years of age in whom they may also cause stomach problems when used in sugar-free medicine if the daily intake is high. Among the bulk sweeteners the most commonly used are sugar alcohols like xylitol, sorbitol, and so forth. Field studies on xylitol, carried out in Russia, Hungary, and Estonia [37], have shown that xylitol is noncariogenic. Moreover, four clinical trials of xylitol in chewing gum have been conducted, namely, Turku chewing-gum study [34], the Ylivieska study [38], the Montreal study [39], and, most recently, the Belize study [40]. All these studies have shown that the use of xylitol helps in the prevention of dental caries. Beside these four chewing-gum studies there is also clinical evidence that xylitol candies are as effective as xylitol gum in caries prevention and that it is economically feasible to include xylitol in school-based caries control programs [41]. The Belize study [40] is the first clinical trial of xylitol that enables the caries-preventive action of xylitol to be compared with sorbitol, and the results indicate that xylitol is superior in reducing caries. These findings should now be validated in randomized studies that account for dietary habits, oral hygiene practice, and socioeconomic status in other populations. Despite the promising findings, there is at present no strong evidence from clinical studies of a superior cariostatic action of xylitol compared with other polyalcohols [42, 43].

## 7. Conclusion

Albeit sugar is associated with the dental diseases like dental caries, we emphasize the fact that sugar alone is not the sole determinant of these diseases. To prevent dental diseases, oral health care workers should persuade their patients to adopt special dietary programs and educate patients and motivate them to alter their customary dietary behavior. Furthermore, such health education must compete with the food manufacturers marketing techniques to significantly reduce dental caries in the population at large.
